# An Inventory Model for Deteriorating Drugs with Stochastic Lead Time

**DOI:** 10.3390/ijerph15122772

**Published:** 2018-12-07

**Authors:** Jian Li, Lu Liu, Hao Hu, Qiuhong Zhao, Libin Guo

**Affiliations:** 1 Research Base of Beijing Modern Manufacturing Development, College of Economics and Management, Beijing University of Technology, Beijing 100124, China; lijiansem@bjut.edu.cn; 2 School of Economics and Management, Beihang University, Beijing 100091, China; 3 Beijing Key Laboratory of Emergency Support Simulation Technologies for City Operations, Beihang University, Beijing 100091, China; 4 Beijing International Science and Technology Cooperation Base for City Safety Operation and Emergency Support, Beihang University, Beijing 100091, China; 5 College of Information Science and Technology, Beijing University of Chemical Technology, Beijing 100029, China; huhao19820922@sina.com (H.H.); glb0412@163.com (L.G.)

**Keywords:** stochastic lead time, deteriorating drugs, inventory model, supply chain education

## Abstract

Inventory management of deteriorating drugs has attracted considerable attention recently in hospitals. Drugs are a kind of special product. Two characteristics of some drugs are the shorter shelf life and high service level. This causes hospitals a great deal of difficulty in inventory management of perishable drugs. On one hand, hospitals should increase the drug inventory to achieve a higher service level. On the other hand, hospitals should decrease the drug inventory because of the short shelf life of drugs. An effective management of pharmaceuticals is required to ensure 100% product availability at the right time, at the right cost, in good conditions to the right customers. This requires a trade-off between shelf-life and service level. In addition, many uncontrollable factors can lead to random lead time of drugs. This paper focuses on deteriorating drugs with stochastic lead time. We have established a stochastic lead time inventory model for deteriorating drugs with fixed demand. The lead time obeyed a certain distribution function and shortages were allowed. This model also considered constraints on service level, stock space and drug shelf life. Through the analysis of the model, the shelf life of drugs and service level were weighted in different lead time distributions. Empirical analysis and sensitivity analysis were given to get reach important conclusions and enlightenment.

## 1. Introduction

Inventory management has been a topic of extensive research in the healthcare sector. The main goal is to reduce the cost of healthcare without sacrificing customer service, and ensure drug quality during storage. Because of China’s medical system reform, drug inventory management is attracting more attention. Barbieri et al. [1] analyzed an updated picture of the evolution in the pharmaceutical sector, and showed positive effects from the process of restructuring of the pharmaceutical sector in China. In this situation, a drug supply guarantee system should be put forward as a very high priority need, as should drug inventory management. Pharmaceutical products are a special kind of product. Expiration or supply shortages may delay the treatment of patients and even endanger their lives. Thus, there are strict requirements for the shelf life and service level of the drug supply. The target of pharmaceutical inventory management is to accelerate turnover of drugs, reduce inventory costs and prevent the occurrence of mistakes.

Raafat [2] showed that drugs are a special kind of deteriorating good with fixed utility. Drug efficacy will diminish over time. According to the expiration rate, the Chinese Pharmaceutical Association has divided drugs into three categories: (1) rapidly expiring drugs, such as the various vaccine injections and so on; (2) drugs with a certain shelf life, where the drug’s efficacy will reduce over its lifetime, such as the vast majority of OTC drugs, etc.; (3) non-expiring drugs, for example, pharmaceutical barium sulfate (barium reagent), other bulk drugs and most Chinese herbal medicines.

Medicines require special consideration in inventory control. Almarsdóttir and Traulsen [3] identified some causes, including the fundamental differences between drugs and other consumer products. For example, medicines are researched, developed, manufactured, and distributed under strict regulatory requirements. Drugs, especially some important commonly used medicines, often require a high service level. Some of them even cannot be in short supply. Drug demand will show sudden changes, increasing suddenly during different seasons or with bursts during a disease epidemic. Losses caused by drug shortages are much higher than the losses of general product shortage. 

We review some of the most relevant literature on medicine inventory in the following sections. The related literature mainly talks about the pharmaceutical inventory management, stochastic lead time, and the shelf life of drugs.

The pharmaceutical product inventory control problem is a difficult one and has been studied extensively under various environments. Uthayakumar and Priyan [4] stated that existing inventory control problems for the perishable products may not be applicable for the pharmaceutical products. Thus it was necessary for controlling pharmaceutical products to build specific inventory model to achieve target customer service level and reduce inventory costs.

Zhu et al. [5] and Priyanand and Uthayakumar [6] established an integrated inventory model for pharmaceutical products in a two-echelon supply chain at minimum supply chain cost. Saedi etc. [7] proposed a stochastic model to find the optimal inventory policy for a healthcare facility to proactively minimize the effect of drug shortages in the presence of uncertain disruptions and demand. Tseng et al. [8] suggested a Gcoin blockchain as the base of the data flow of drugs to create transparent drug transaction data. Additionally, the regulation model of the drug supply chain could be altered from the inspection and examination only model to the surveillance net model. In this paper, we consider pharmaceutical inventory management with stochastic lead time.

As we have known that, lead time has a strong effect on inventory management and ordering strategy. And it is often uncertain in practices. Many scholars study the inventory problem with stochastic lead time.

Shibuya et al. [9] and Hariga and Ben-Daya [10] analyzed lead time reduction inventory models which are based on the continuous review inventory system. Hoque [11,12] developed a manufacturer–buyer integrated inventory model and a vendor–buyer integrated production–inventory model with a normal distribution of lead times. Louly and Dolgui [13] dealt with Material Requirement Planning (MRP) software parameterization under uncertainly lead time. Isotupa and SamantaIn [14] analyzed a lost sales (s, Q) inventory system with two types of customers and stochastic lead time. Das and Hanaoka [15] developed a humanitarian disaster relief inventory model that assumed a uniformly distributed function in both lead-time and demand parameters, which were appropriate considering the limited historical data on relief operation. Heydari [16,17] investigated the issue of the upstream stochastic lead time in a two-stage supply chain with both the supplier and the retailer with stochastic lead time or retailer stochastic lead time. Rong and Maiti [18] illustrated an Economic Order Quantity (EOQ) inventory model with fuzzy stochastic demand and controllable lead-time. Lin [19] dealt with investing in lead-time variability reduction problems for the integrated vendor–buyer supply chain system. Heydari et al. [20] analyzed the destructive effects of upstream aggregated stochastic lead time on the supply chain performance. Disney et al. [21] studied the impact of stochastic lead times with order crossover on inventory costs and safety stocks in the order-up-to policy. Song et al. [22] considered a single-product, two-source inventory system with Poisson demand and stochastic lead times. This paper studies the effects of different distributions subject to lead time for pharmaceutical inventory management.

Besides the stochastic lead time discussed above, the shelf life of products is also one of the issues that inventory management considered. It will reduce the product inventory or cause losses when the stock fails. Therefore more and more scholars have began to consider the impact of shelf life for the inventory problem in the inventory model.

Yan et al. [23] focused on the determination of a feasible production schedule where the products have a limited post-production shelf life. Haijema [24] considered perishables which are characterized by a short maximum shelf life. A new class of stock-level dependent ordering policies was presented. Kouki et al. [25] considered a perishable inventory system that operates under stochastic demand, constant lifetime and a constant lead time. Wu and Zhao [26] considered the fixed demand goods with its shelf life. And from the point of view of production operation, they studied the supply chain joint ordering decision on based of commercial credit. Muriana [27] presented a mathematical stochastic model for perishable open-dating foods including shortage and outdating costs.

In summary, the studies are divided into two categories. One is to take general drugs into consideration. In these works, demand or lead time is stochastic to fit the actual situation without considering shelf life. Most studies are based on the EOQ model which can be approximately used to solve general drug inventory ordering policy issues. The other involves drug shelf life. Based on deteriorating inventory model, the works use constant or random deterioration rates to reflect drug shelf life. However, in common life perishable drugs have a fixed shelf life.

At present, most hospitals use professional computer software management and manual management measures to manage the hospital’s drug stocks. They control the validity of the drug by inputting drug shelf life into the software and regular inspections. This way only can reduce the medical accidents caused by the misuse of the expired medicines, but not to reduce the losses caused by the failure of the medicines because of the backlog of stocks. In view of this, we consider the drug shelf life from the beginning of the order. We develop a reasonable order quantity and ordering cycle through the demand, lead time and other factors. We weigh the shelf life of drugs and service level for different distributions of lead time to reduce drug inventory costs and drug failure costs.

In this paper, what we address is the liquid pharmaceutical preparations inventory problem of a hospital. First, we establish a pharmaceutical inventory model with shelf life and service level constraints with a stochastic lead time. Then, we obtain the optimal order quantities and reorder points by using data from some hospitals in weighting the shelf life of drugs and service level. These verify the efficiency of our model. Our model is more suitable for perishable drugs with a fixed shelf life and stochastic lead time. Finally, we conduct a sensitivity analysis for this model to provide some insights for medicine inventory management. A conclusion can be drawn that distribution which follows a certain lead time has a certain influence on total cost, reordering point and ordering quantity. Especially, shelf life has a different degree of influence on drug ordering lot sizes and total cost in different distributions which follow a lead time.

The remainder of this paper is organized as follows: in the next section, we introduce the notations and assumptions, and then mathematically formulate a drug inventory model with stochastic lead time; and further, we obtain the optimal solution with different lead time distributions. Section 3 provides numerical examples to demonstrate the results of the proposed model. Section 4 provides a sensitivity analysis of shelf life to analyze the impact of shelf life on ordering lot sizes and total inventory cost. Finally, Section 5 concludes the paper.

## 2. Basic Model

### 2.1. Notations

The model is built by considering the following notation:

Q: Ordering lot sizes for a drug;
*L*: Lead time for a drug;
D: Average demand for a drug per year;
h: Holding cost per unit per year for a drug;
A: Fixed ordering cost per order for a drug;
*K*: Ordering cost per unit for a drug;
*C*: Shortages cost per unit for a drug;
1−α: Fraction of demands for a drug that are not met from stock, i.e., the service level;
f: Storage space for a drug;
S: Shelf life for a drug;
$T_{s}$: Expiration date for a drug;
*W*: Total space available for all drugs;
*T*: Replenishment cycle;
*r*: Reordering point;
*I(t*): Inventory levels;
*g(t*): Probability density function of lead time.

### 2.2. Assumptions

The model is built by considering the following assumptions:

(1) For a drug, its average demand per year is a fixed constant;
(2) For a drug, the hospital uses a continuous review policy and the order quantity Q is placed when its inventory level falls below the reorder point r;
(3) For a drug, lead time L follows a probability distribution function *g(t*);
(4) Shortages are allowed, but will cause a shortage cost. Replenishment is not needed;
(5) First Input First Output (FIFO) principle is used;
(6) For a drug, hospital requires that the drug remaining expiration date should be more than the drug shelf life in ordering.

### 2.3. Basic Model

**Figure 1:**
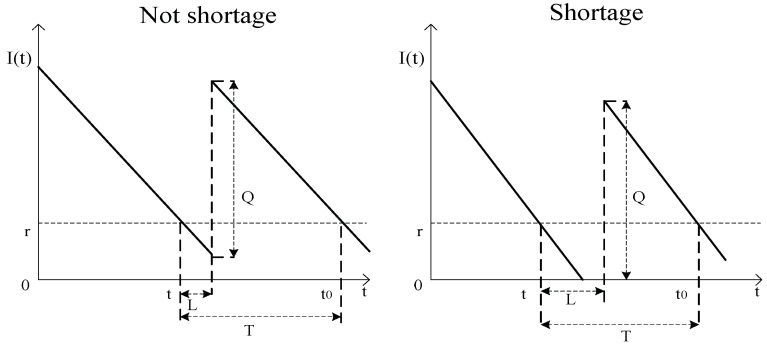
Inventory level.

Figure 1 shows that the inventory level is maximum at *t* = 0 and it depletes over time until it drops to r and new drugs are ordered. Because of the lead time variability, two distinct cases should be considered.

#### 2.3.1. Storage Costs

Storage costs consist of two parts which are shown in Figure 1. One is storage costs caused during lead time (*t*, *t* + *L*). The other is storage costs caused during the period from drug arrival to the next order, i.e., (*t* + *L*, *t*_0_). We assume that *Y* is the stock number in cycle time. Then we can get two cases of the relationship between *Y* and *L*:

$Y_{1}$ denotes inventory in lead time:

(1) $$Y_{1}=\{\begin{array}{c} \frac{\left(2r-DL\right)L}{2}\left(0\leq L<\frac{r}{D}\right)\\ \frac{r^{2}}{2D}\left(\frac{r}{D}\leq L\leq \frac{W}{fD}\right) \end{array}$$

so, the expected storage cost in (*t*, *t* + *L*) is:

(2) $$E\left(C_{Y_{1}}\right)=h\int_{0}^{\frac{r}{D}}\frac{\left(2r-Dt\right)t}{2}g\left(t\right)dt+h\int_{\frac{r}{D}}^{\frac{W}{fD}}\frac{r^{2}}{2D}g\left(t\right)dt$$

$Y_{2}$ denotes inventory in the period from drug arrival to the next order

(3) $$Y_{2}=\{\begin{array}{c} \frac{\left(Q+2r-DL\right)\left(Q-DL\right)}{2D}\left(0\leq L<\frac{r}{D}\right)\\ \frac{Q^{2}-r^{2}}{2D}\left(\frac{r}{D}\leq L\leq \frac{W}{fD}\right) \end{array}$$

So we get expected storage costs in (*t* + *L*, *t*_0_) given by:

(4) $$E\left(C_{Y_{2}}\right)=h\int_{0}^{\frac{r}{D}}\frac{\left(Q+2r-DL\right)\left(Q-DL\right)}{2D}g\left(t\right)dt+h\int_{\frac{r}{D}}^{\frac{W}{fD}}\frac{Q^{2}-r^{2}}{2D}g\left(t\right)dt$$

In ordering cycle time *T*, the expected total storage costs are:

(5) $$E\left(C_{Y}\right)=E\left(C_{Y_{1}}\right)+E\left(C_{Y_{2}}\right)$$

Hence using Equations (2), (4), (5), we have:

(6) $$E\left(C_{Y}\right)=h\int_{0}^{\frac{r}{D}}\frac{\left(2r-Dt\right)t}{2}g\left(t\right)dt+h\int_{\frac{r}{D}}^{\frac{W}{fD}}\frac{r^{2}}{2D}g\left(t\right)dt+h\int_{0}^{\frac{r}{D}}\frac{\left(Q+2r-Dt\right)\left(Q-Dt\right)}{2D}g\left(t\right)dt\;+h\int_{\frac{r}{D}}^{\frac{W}{fD}}\frac{Q^{2}-r^{2}}{2D}g\left(t\right)dt$$

#### 2.3.2. Shortage Costs

Figure 1 shows that an out of stock situation happened during the lead time. From Figure 1, the amount of shortages can be expressed by the area surrounded by the inventory horizontal line and the timeline in the lead time. We assume that *Z* is the amount of shortage in the lead time. Then we can get two cases of the relationship between *Z* and *L*:

(7) $$Z=\{\begin{array}{c} 0\;\left(0\leq L\leq \frac{r}{D}\right)\\ \frac{{\left(DL-r\right)}^{2}}{2D}\left(\frac{r}{D}\leq L\leq \frac{W}{fD}\right) \end{array}$$

so we get expected shortage costs in lead time given by:

(8) $$E\left(C_{Z}\right)=C\int_{\frac{r}{D}}^{\frac{W}{fD}}\frac{{\left(Dt-r\right)}^{2}}{2D}g\left(t\right)dt$$

#### 2.3.3. Ordering Costs

Ordering costs include fixed ordering costs and variable ordering costs. We assume that *A* is the fixed ordering costs and *KQ* is the variable ordering costs, so we get the expected ordering costs:

(9) $$E\left(C_{0}\right)=A+KQ$$

#### 2.3.4. Ordering Cycle Time

In this model we assume that ordering cycle time *T* is a period from one ordering point to the next point. In this period, the amount of ordering *Q* and demand *D* cause a change of inventory, so the ordering cycle time *T* is:

(10) $$T=\frac{Q}{D}$$

#### 2.3.5. Reordering Point

Reordering point is the inventory at the time of replenishment. According to the definition of first category service level, the probability of enough inventory is given by:

(11) $$\alpha =\int_{0}^{\frac{r}{D}}g\left(t\right)dt$$

We consider that the hospital requires a high service level with service level constraint given by:

(12) $$\alpha =\int_{0}^{\frac{r}{D}}g\left(t\right)dt\geq 98\%$$

#### 2.3.6. Shelf Life

In this paper, we assume that drug shelf life in ordering should be more than the ordering cycle time, so the shelf life constraint is:

(13) $$\int_{0}^{S-\frac{Q}{D}}g\left(t\right)dt\geq 99\%$$

For the total inventory costs we consider storage costs, shortage costs and ordering costs in an ordering cycle time. Hence, expected total cost per cycle is:

(14) $$E\left[C\left(Q,r\right)\right]=\frac{E\left(C_{0}\right)+E\left(C_{Y}\right)+E\left(C_{Z}\right)}{T}$$

Using Equations (6), (8), (9) we can transform Equation (13) into:

(15) $$\begin{array}{l} E\left[C\left(Q,r\right)\right]\\ =\frac{\begin{array}{c} A+KQ+h\int_{0}^{\frac{r}{D}}\frac{\left(2r-Dt\right)t}{2}g\left(t\right)dt+h\int_{\frac{r}{D}}^{\frac{W}{fD}}\frac{r^{2}}{2D}g\left(t\right)dt+h\int_{0}^{\frac{r}{D}}\frac{\left(Q+2r-Dt\right)\left(Q-Dt\right)}{2D}g\left(t\right)dt\\ +h\int_{\frac{r}{D}}^{\frac{W}{fD}}\frac{Q^{2}-r^{2}}{2D}g\left(t\right)dt+C\int_{\frac{r}{D}}^{\frac{W}{fD}}\frac{{\left(Dt-r\right)}^{2}}{2D}g\left(t\right)dt \end{array}}{T}\\ =\frac{DA+C\int_{\frac{r}{D}}^{\frac{W}{fD}}\frac{{\left(r-Dt\right)}^{2}}{2}g\left(t\right)dt}{Q}+KD+\frac{hQ}{2}\int_{0}^{\frac{W}{fD}}g\left(t\right)dt-hD\int_{0}^{\frac{r}{D}}tg\left(t\right)dt+h\int_{0}^{\frac{r}{D}}rg\left(t\right)dt \end{array}$$

Now our problem is to find the optimal *Q* and *r* in a production run that minimizes the integrated expected total cost expressed by Equation (14). The result also satisfies the service level constraint expressed by Equation (11), the shelf life constraint expressed by Equation (12) and the ordering lot sizes constraint. In other words, the problem of a hospital’s deteriorating drugs inventory system involving uncertain lead time can be mathematically formulated as the following nonlinear programming model:

Min Z=E[C(Q,r)]

(16) $$s.t.\{\begin{array}{c} \alpha =\int_{0}^{\frac{r}{D}}g\left(t\right)dt\geq 98\%\\ \int_{0}^{S-\frac{Q}{D}}g\left(t\right)dt\geq 99\%\\ Q\geq r\\ Q,r\geq 0 \end{array}$$

### 2.4. The Lead Time Follows Uniform Distribution or Exponential Distribution

In this section, we discuss two cases of the drug inventory model with lead times following different distributions.

**Proposition** **1.** *When the lead time follows a uniform distribution* $g\left(t\right)=\frac{1}{b-a}(a\leq t\leq b)$ *, the inventory model can be written as follows:*

$$Min\;Z=E\left[C\left(Q,r\right)\right]=\frac{DA+\frac{C}{2\left(b-a\right)}\left[r^{2}b-\frac{r^{3}}{3D}-rDb^{2}+\frac{b^{3}D^{2}}{3}\right]}{Q}+KD+\frac{hQ}{2}+\frac{hr^{2}+hD^{2}a^{2}-2Dahr}{2\left(b-a\right)D}$$

(17) $$s.t.\{\begin{array}{c} \alpha =\int_{a}^{\frac{r}{D}}\frac{1}{b-a}dt\geq 98\%\\ b+\frac{Q}{D}\leq S\\ Q\geq r\\ 0\leq a\leq \frac{r}{D}\leq b\leq \frac{W}{fD}\\ Q,r,t\geq 0 \end{array}$$

We obtain the optimal value of *Q* as follows:

(18) $$Q^{*}=\sqrt{\frac{2DA+\frac{C}{\left(b-a\right)}\left[r^{2}b-\frac{r^{3}}{3D}-rDb^{2}+\frac{b^{3}D^{2}}{3}\right]}{h}}$$

The proof is provided in the Appendix.

**Proposition** **2.** *When the lead time follows an exponential distribution* $g\left(t\right)=\{\begin{array}{c} \lambda e^{-\lambda t}\;t>0\\ 0\;t\leq 0 \end{array}\;(\lambda >0)$ *, the inventory model can be written as follows:*

$$\begin{array}{l} Min\;Z=E\left[C\left(Q,r\right)\right]\\ \;=\frac{AD}{Q}+KD\\ \;+\left(\frac{DWC^{2}}{\lambda fQ}-\frac{2rDC^{2}}{\lambda Q}+\frac{C^{2}D^{2}}{Q\lambda^{2}}-\frac{rWC^{2}}{fQ}+\frac{C^{2}r^{2}}{Q}+\frac{C^{2}W^{2}}{2Qf^{2}}\right)\left(e^{-\frac{\lambda r}{D}}-e^{-\frac{\lambda W}{fD}}\right)\\ \;+\frac{hQ}{2}\left(1-e^{-\frac{\lambda W}{fD}}\right)-\frac{hD}{\lambda }\left(1-e^{-\frac{\lambda r}{D}}\right)+hr \end{array}$$

(19) $$s.t.\{\begin{array}{c} \begin{array}{c} 98\%\leq \int_{0}^{\frac{r}{D}}\lambda e^{-\lambda t}dt\leq 99\% \end{array}\\ \int_{0}^{S-\frac{Q}{D}}\lambda e^{-\lambda t}dt\geq 99\%\\ Q\geq r\\ Q,r,t\geq 0 \end{array}$$

We obtain the optimal value of *Q* as follows:

(20) $$Q^{*}=\sqrt{\frac{2DA+2C^{2}\left(\frac{DW}{\lambda f}-\frac{2rD}{\lambda }+\frac{D^{2}}{\lambda^{2}}-\frac{rW}{f}+r^{2}+\frac{W^{2}}{2f^{2}}\right)\left(e^{-\frac{\lambda r}{D}}-e^{-\frac{\lambda W}{fD}}\right)}{h\left(1-e^{-\frac{\lambda W}{fD}}\right)}}$$

The proof is provided in the Appendix.

From the above we can find that the object function of the inventory model has changed when the lead time follows a different distribution. In the next section, we will compare different optimal solutions when the lead time follows different distributions by numerical analysis.

## 3. Numerical Analysis

### 3.1. The Lead Time Follows Uniform Distribution

In this section, numerical analysis is conducted to validate the inventory model. The parameters are listed in Table 1. The lead time is assumed to follow a uniform distribution with a probability density function given by:

$$g\left(t\right)=\frac{1}{0.03}\;\left(0.01\leq t\leq 0.04\right)$$

Mean lead time is assumed to be 9 days, so, $E\left(L\right)=\frac{a+b}{2}=0.025$, $D\left(L\right)=\frac{{\left(b-a\right)}^{2}}{12}=0.000075$.

Then we have the following results:

$$\begin{array}{ll} Min\;Z=E\left[C\left(Q,r\right)\right] & =\frac{DA+\frac{C}{2\left(b-a\right)}\left[r^{2}b-\frac{r^{3}}{3D}-rDb^{2}+\frac{b^{3}D^{2}}{3}\right]}{Q}+KD+\frac{hQ}{2}+\frac{hr^{2}+hD^{2}a^{2}-2Dahr}{2\left(b-a\right)D}\\ & =\frac{12,000+16,666.67\times \left(0.04r^{2}-\frac{r^{3}}{1800}-0.96r+7.68\right)}{Q}+300,000+2Q\\ & +\frac{4r^{2}-48r+144}{36} \end{array}$$

$$s.t.\{\begin{array}{c} \int_{0.01}^{\frac{r}{600}}33.33dt\geq 98\%\\ 0.04+\frac{Q}{600}\leq 0.25\\ Q\geq r\\ 6\leq r\leq 24\\ Q,r\geq 0 \end{array}$$

According to simplified formulas, with the above simplified formula, we have:

$$\begin{array}{l} Min\;Z=E\left[C\left(Q,r\right)\right]\\ \;=\frac{12,000+16,666.67\times \left(0.04r^{2}-\frac{r^{3}}{1800}-0.96r+7.68\right)}{Q}+300,000+2Q\\ \;+\frac{4r^{2}-48r+144}{36} \end{array}$$

$$s.t.\{\begin{array}{c} Q\leq 126\\ Q\geq r\\ 23.58\leq r\leq 24\\ Q,r\geq 0 \end{array}$$

**Figure 2:**
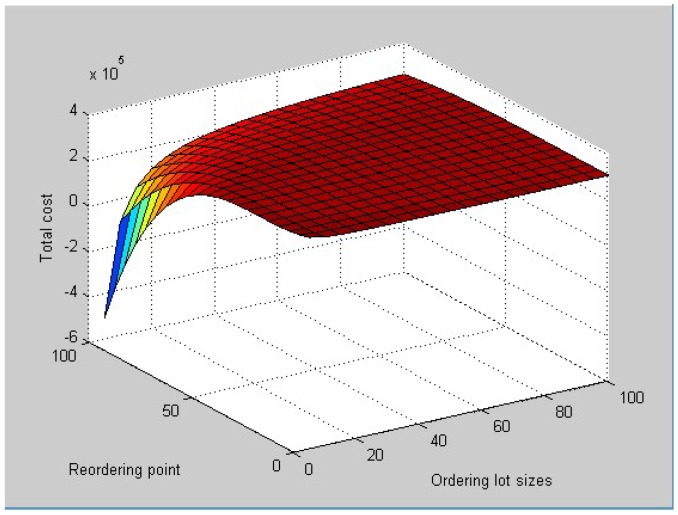
Relationship between Z, Q, and r.

Figure 2 shows the relationship between *Z*, *Q*, and *r*. Then we obtain the optimal ordering lot sizes *Q* = 77.46 packages, reordering point *r* = 23.58 packages, ordering cycle time *T* = 47.12 days, and expected total costs E[C(Q,r)] = 300,344.19 yuan.

### 3.2. The Lead Time Follows Exponential Distribution

In this section, numerical analysis is conducted to validate the inventory model. Parameters are listed in Table 2. The lead time is assumed to follow an exponential distribution with a probability density function given by:

$$g\left(t\right)=\{\begin{array}{c} 40e^{-40t}\;t>0\\ 0\;t\leq 0 \end{array}\;(\lambda >0)$$

The mean the lead time is assumed to be 9 days. λ=40, so, $E\left(L\right)=\frac{1}{\lambda }=0.025$, $D\left(L\right)=\frac{1}{\lambda }=0.025$.

Then we have the following results:

$$\begin{array}{l} Min\;Z=E\left[C\left(Q,r\right)\right]\\ \;=\frac{12,000}{Q}+300,000\\ \;+\left(\frac{1.66\times 10^{10}}{Q}-\frac{1.97\times 10^{8}r}{Q}+\frac{10^{6}r^{2}}{Q}\right)\left(e^{-\frac{r}{15}}-e^{-\frac{100}{9}}\right)+2Q\left(1-e^{-\frac{100}{9}}\right)\\ \;-60\left(1-e^{-\frac{r}{15}}\right)+4r \end{array}$$

$$s.t.\{\begin{array}{c} 58.68\leq r\leq 69.08\\ Q\leq 130.92\\ Q\geq r\\ Q,r\geq 0 \end{array}$$

**Figure 3:**
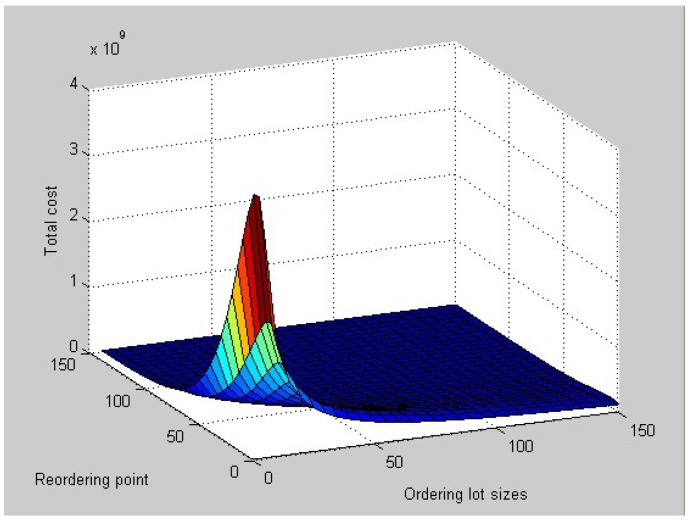
Relationship between *Z*, *Q*, and *r*.

Figure 3 shows the relationship between *Z*, *Q*, and *r*. Then we obtain the optimal ordering lot sizes *Q* = 130.92 packages, reordering point *r* = 69.08 packages, ordering cycle time *T* = 79.6 days, and expected total costs E[C(Q,r)] = 892,566.86 yuan.

From the numerical analysis solutions and figures above, we can draw the conclusion that ordering lot sizes and reordering point cause different changes to the total inventory cost when the lead time follows different distributions. When the lead time follows a uniform distribution, that is for each member of the family, all intervals of the same length on the distribution’s support are equally probable, from the corresponding figure, the total inventory cost changes with the changing of ordering lot sizes and reordering point and the magnitude of the change is large. When the lead time follows an exponential distribution, which is a process in which events occur continuously and independently at a constant average rate, from its figure, the total inventory cost changes with the changing of ordering lot sizes and reordering point, but the magnitude of the change is large at the beginning and then tends to become gentle.

## 4. Sensitivity Analysis of Shelf Life for a Drug

### 4.1. The Lead Time Follows a Uniform Distribution

Consider the constraints first. Other parameters do not change while shelf life *S* changes from 0.02 year to 0.33 year. That is:

$$s.t.\{\begin{array}{c} 48.75\leq r\leq 49.2\\ Q\leq -49.2+600\times S\\ Q\geq r\\ Q,r\geq 0 \end{array}$$

When *S* changes, we can get different *r* and *Q* which can minimize the total costs. Table 3 shows the experimental data.

**Figure 4:**
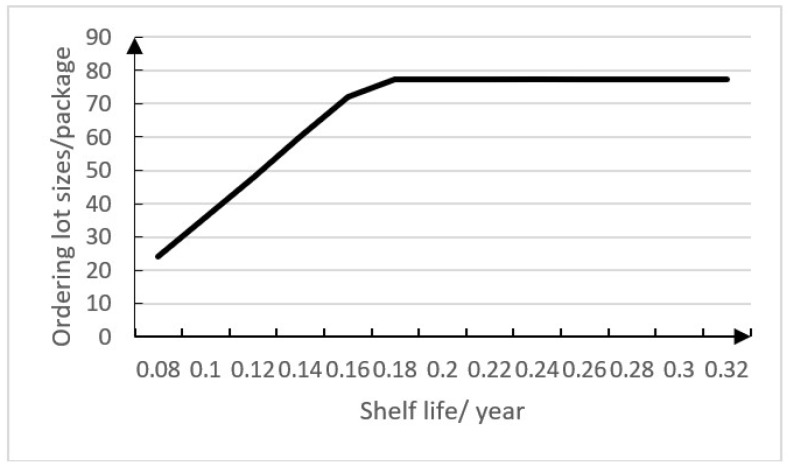
Relationship between ordering lot sizes (*Q*) and shelf life (*S*).

Figure 4 shows that ordering lot sizes changes with the changing shelf life. From Figure 4, we can know the ordering lot size will increase 100% when the shelf life increases 100% within a certain range, so shelf life has a certain sensitivity to ordering lot size within a certain range because ordering lot size is made a constraint by shelf life. The hospital can increase ordering lot sizes to make sure that could use all the drugs during their shelf life, when the shelf life for a drug is long, and the shelf life of a drug has little influence on its ordering lot size when the shelf life has a certain duration.

**Figure 5:**
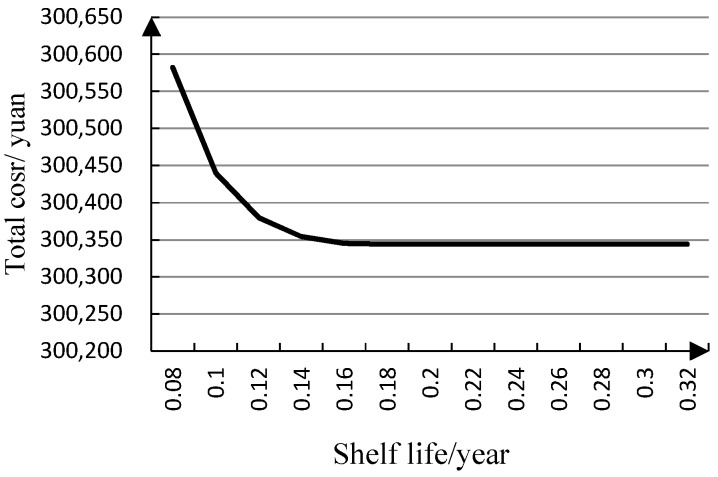
Relationship between total cost (*TC*) and shelf life (*S*).

Figure 5 shows that total inventory cost decreases fast first and then gently with increasing shelf life.

From Figure 5, we can know that the total inventory cost will not decrease 100% when the shelf life increases 100%. Shelf life has little sensitivity to total inventory cost. Because total inventory cost is affected by ordering lot sizes, the hospital should increase the number of orders to meet drug demand, when the shelf life of a drug is short. Then the total inventory cost changes gently, because ordering lot sizes change gently when the shelf life for a drug is long, so the shelf life for a drug has an influence on the total inventory cost.

### 4.2. The Lead Time Follows an Exponential Distribution

Consider the constraints first. Other parameters do not change while shelf life *S* changes from 0.02 year to 0.48 year. That is:

$$s.t.\{\begin{array}{c} 58.68\leq r\leq 69.08\\ Q\leq -69.08+600\times S\\ Q\geq r\\ Q,r\geq 0 \end{array}$$

When *S* changea, we can get different *r* and *Q* which can minimize the total costs. Table 4 shows the experimental data.

**Figure 6:**
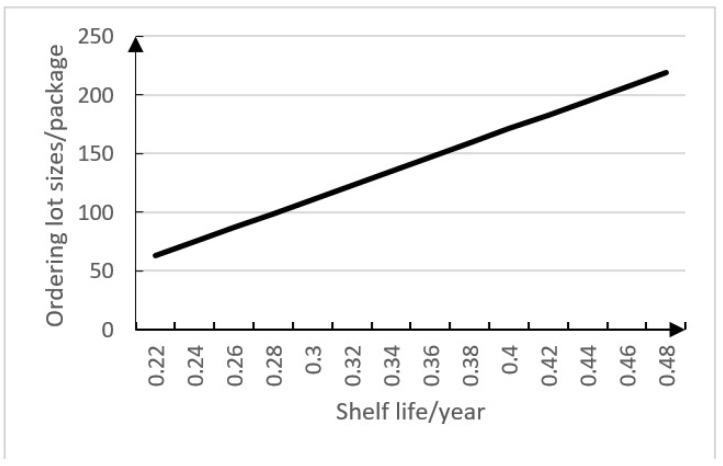
Relationship between ordering lot sizes (*Q*) and shelf life (*S*).

Figure 6 shows that ordering lot sizes increases with increasing shelf life. From Figure 6, we can know the ordering lot size will increase 100% when the shelf life increases 100%, so shelf life has a certain sensitivity to ordering lot size because the ordering lot size is made a constraint by shelf life. The hospital should reduce ordering lot sizes to make sure that could use all drugs during their shelf life, when the shelf life for a drug is short, so theshelf life for a drug has an influence on its ordering lot sizes.

**Figure 7:**
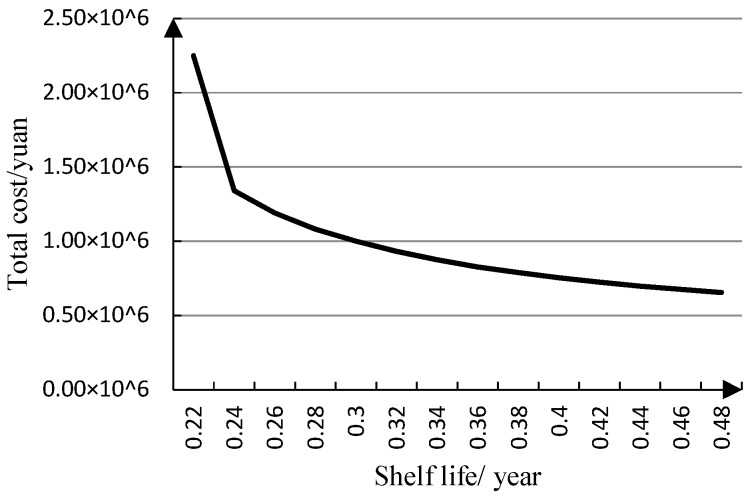
Relationship between total cost (*TC*) and shelf life (*S*).

Figure 7 shows that total inventory cost decreases fast first and then gently with increasing shelf life. From Figure 7, we can know that the total inventory cost will decrease 100% when the shelf life increases 100%, so shelf life has a certain sensitivity to total inventory cost because the total inventory cost is affected by ordering lot sizes. The hospital should increase the number of orders to meet drug demand, when the shelf life of a drug is short. Then the total inventory cost decreases gently, because the holding cost increases when the shelf life of a drug is long, so the shelf life for a drug has an influence on total inventory cost.

From the sensitivity analysis solutions and the figures above, we can draw the conclusion that shelf life has different degrees of influence on ordering lot sizes and total cost when the lead time follows different distributions. If the lead time follows a uniform distribution, from the corresponding figure, shelf life has a great effect on ordering lot sizes and total cost in a relatively short time horizon. If the lead time follows an exponential distribution, its figure shows that shelf life has a great effect on ordering lot sizes and total cost in a relatively long time horizon. 

## 5. Conclusions

Inventory management is one of the most challenge activities for healthcare organizations and it has recently began to be taken seriously by healthcare managers. In a fiercely competitive environment, inventory managers are interested in deteriorating drugs. However, decision making in drug inventory management is often uncertain. We know that the uncertainty of external factors leads to uncertainty of lead time in real life. This study took the hospital inventory with stochastic lead time into consideration. More liquid pharmaceutical preparations are being used in hospitals. These kinds of drugs have an unstable nature with a short shelf life, so this the starting point of this work where we discuss these drugs’ inventory management problems.

This research established a drug inventory model where the objective function is total cost minimization per unit time. Firstly, we took the actual situation of inventory space, short shelf life of liquid pharmaceutical preparations and high service level requirements of drug into consideration to propose constraints such as inventory space limitations, shelf life limitations, and service level limitations. Secondly, we assumed that lead time followed a uniform distribution or an exponential distribution, and compared with these two models. Thirdly, this paper took the relevant data of a hospital into the model to obtain the optimal reordering point, the optimal ordering lot sizes and optimal ordering cycle in weighting the shelf life of drugs and service level. Then we performed a sensitivity analysis of the total cost within the constraints. We can draw a conclusion that in a distribution system which lead time scenario is followed has a certain influence on total cost, reordering point and ordering quantity. Especially, shelf life has a different degree of influence on drug ordering lot sizes and total cost in different distributions which followed lead times. Based on the traditional drug inventory problem, this work took drug shelf life, service level and stochastic lead time into consideration, which is more appropriate for the actual situation. In future studies, uncertainty of drug demand should be considered. Besides to fit with the actual situation of hospitals, we should extend this research a variety of drugs.

## Figures and Tables

**Table 1:** Parameters of the inventory model.

Parameter	Value	Parameter	Value
*L*	G(t) year	*C*	1000 yuan/package
*D*	600 package/year	*f*	0.3 m^3^/package
*h*	4 yuan/package year	*S*	1/4 year
*A*	20 yuan/lot	*T_S_*	1/2 year
*K*	500 yuan/package	*W*	50 m^3^

**Table 2:** Parameters of the inventory model.

Parameter	Value	Parameter	Value
*L*	G(t) year	*C*	1000 yuan/package
*D*	600 package/year	*f*	0.3 m^3^/package
*h*	4 yuan/package year	*S*	1/3 year
*A*	20 yuan/lot	*T_S_*	1/2 year
*K*	500 yuan/package	*W*	50 m^3^

**Table 3:** Relevant data for the sensitivity analysis.

*S*	*Q*	*r*	*TC*
0.08	24.0000	23.58	300,582.37
0.10	36.0000	23.58	300,439.69
0.12	48.0000	23.58	300,380.35
0.14	60.0000	23.58	300,354.35
0.16	72.0000	23.58	300,345.02
0.18	77.4619	23.58	300,344.19
0.20	77.4617	23.58	300,344.19
0.22	77.4623	23.58	300,344.19
0.24	77.4625	23.58	300,344.19
0.26	77.4616	23.58	300,344.19
0.28	77.4612	23.58	300,344.19
0.30	77.4615	23.58	300,344.19
0.32	77.4623	23.58	300,344.19

**Table 4:** Relevant data of the sensitivity analysis.

*S*	*Q*	*r*	*TC*
0.22	62.92	62.92	2.25 × 10 ^6^
0.24	74.92	69.08	1.34 × 10 ^6^
0.26	86.92	69.08	1.19 × 10 ^6^
0.28	98.92	69.08	1.08 × 10 ^6^
0.3	110.92	69.08	9.99 × 10 ^5^
0.32	122.92	69.08	9.31 × 10 ^5^
0.34	134.92	69.08	8.75 × 10 ^5^
0.36	146.92	69.08	8.28 × 10 ^5^
0.38	158.92	69.08	7.88 × 10 ^5^
0.4	170.92	69.08	7.54 × 10 ^5^
0.42	182.92	69.08	7.24 × 10 ^5^
0.44	194.92	69.08	6.98 × 10 ^5^
0.46	206.92	69.08	6.75 × 10 ^5^
0.48	218.92	69.08	6.55 × 10 ^5^

## Appendix A

Proposition 1 can be proved as follows:
**Proof** **of Proposition 1.** In the following, we assume that the lead time follows Uniform distribution. That is:

(A1) $$g\left(t\right)=\frac{1}{b-a}(a\leq t\leq b)$$

and:

$$0\leq a\leq \frac{r}{D}\leq b\leq \frac{W}{fD}$$

Then, we can transform Equation (15) into the following:

$$\begin{array}{l} Min\;Z=E\left[C\left(Q,r\right)\right]\\ \;=\frac{DA+C\int_{\frac{r}{D}}^{\frac{W}{fD}}\frac{{\left(r-Dt\right)}^{2}}{2}g\left(t\right)dt}{Q}+KD+\frac{hQ}{2}\int_{0}^{\frac{W}{fD}}g\left(t\right)dt-hD\int_{0}^{\frac{r}{D}}tg\left(t\right)dt\\ \;+h\int_{0}^{\frac{r}{D}}rg\left(t\right)dt\\ \;=\frac{DA+C\int_{\frac{r}{D}}^{b}\frac{{\left(r-Dt\right)}^{2}}{2}\frac{1}{b-a}dt}{Q}+KD+\frac{hQ}{2}\int_{a}^{b}\frac{1}{b-a}dt-hD\int_{a}^{\frac{r}{D}}t\frac{1}{b-a}dt\\ \;+h\int_{a}^{\frac{r}{D}}r\frac{1}{b-a}dt\\ \;=\frac{DA+\frac{C}{2\left(b-a\right)}\left[r^{2}b-\frac{r^{3}}{3D}-rDb^{2}+\frac{b^{3}D^{2}}{3}\right]}{Q}+KD+\frac{hQ}{2}\\ \;+\frac{hr^{2}+hD^{2}a^{2}-2Dahr}{2\left(b-a\right)D} \end{array}$$

(A2) $$s.t.\{\begin{array}{c} \alpha =\int_{a}^{\frac{r}{D}}\frac{1}{b-a}dt\geq 98\%\\ b+\frac{Q}{D}\leq S\\ Q\geq r\\ 0\leq a\leq \frac{r}{D}\leq b\leq \frac{W}{fD}\\ Q,r,t\geq 0 \end{array}$$

(a) Solution This model can be expressed by:

$$Min\;Z=E\left[C\left(Q,r\right)\right]=\frac{DA+\frac{C}{2\left(b-a\right)}\left[r^{2}b-\frac{r^{3}}{3D}-rDb^{2}+\frac{b^{3}D^{2}}{3}\right]}{Q}+KD+\frac{hQ}{2}+\frac{hr^{2}+hD^{2}a^{2}-2Dahr}{2\left(b-a\right)D}$$

(A3) $$s.t.\{\begin{array}{c} \alpha =\int_{a}^{\frac{r}{D}}\frac{1}{b-a}dt\geq 98\%\\ b+\frac{Q}{D}\leq S\\ Q\geq r\\ 0\leq a\leq \frac{r}{D}\leq b\leq \frac{W}{fD}\\ Q,r,t\geq 0 \end{array}$$

The first derivative of total costs with respect to ordering quantity is:

(A4) $$\frac{\partial E\left[C\left(Q,r\right)\right]}{\partial Q}=-\frac{DA+\frac{C}{2\left(b-a\right)}\left[r^{2}b-\frac{r^{3}}{3D}-rDb^{2}+\frac{b^{3}D^{2}}{3}\right]}{Q^{2}}+\frac{h}{2}$$

With the first order condition, we obtain the optimal value of *Q* as follows:

(A5) $$-\frac{DA+\frac{C}{2\left(b-a\right)}\left[r^{2}b-\frac{r^{3}}{3D}-rDb^{2}+\frac{b^{3}D^{2}}{3}\right]}{Q^{2}}+\frac{h}{2}=0$$

(A6) $$Q^{*}=\sqrt{\frac{2DA+\frac{C}{\left(b-a\right)}\left[r^{2}b-\frac{r^{3}}{3D}-rDb^{2}+\frac{b^{3}D^{2}}{3}\right]}{h}}$$

If $0<Q<Q^{*}$, $\frac{\partial E\left[C\left(Q,r\right)\right]}{\partial Q}<0$, E[C(Q,r)] is monotonically decreasing. If $Q>Q^{*}$, $\frac{\partial E\left[C\left(Q,r\right)\right]}{\partial Q}>0$, E[C(Q,r)] is monotonically increasing, so $Q^{*}$ is minimal value of E[C(Q,r)]. Next, we take the first derivative of total costs with respect to reordering point:

(A7) $$\begin{array}{ll} \frac{\partial E\left[C\left(Q,r\right)\right]}{\partial r} & =\frac{C\left(2rb-\frac{r^{2}}{D}-Db^{2}\right)}{2Q\left(b-a\right)}+\frac{rh-Dah}{\left(b-a\right)D}\\ & =-\frac{Cr^{2}}{2QD\left(b-a\right)}+\left[\frac{Cb}{Q\left(b-a\right)}+\frac{h}{D\left(b-a\right)}\right]r-\frac{CDb^{2}}{2Q\left(b-a\right)}-\frac{ah}{b-a} \end{array}$$

Similarly, we obtain the optimal value of r as follows:

(A8) $$-\frac{Cr^{2}}{2QD\left(b-a\right)}+\left[\frac{Cb}{Q\left(b-a\right)}+\frac{h}{D\left(b-a\right)}\right]r-\frac{CDb^{2}}{2Q\left(b-a\right)}-\frac{ah}{b-a}=0$$

Besides:

(A9) $$\begin{array}{c} \Delta =b^{2}-4ac={\left[\frac{Cb}{Q\left(b-a\right)}+\frac{h}{D\left(b-a\right)}\right]}^{2}-4\times \frac{Cr^{2}}{2QD\left(b-a\right)}\times \frac{CD^{2}b^{2}+2ahDQ}{2QD\left(b-a\right)}\\ =\frac{2ChDQ\left(b-a\right)+h^{2}Q^{2}}{Q^{2}D^{2}{\left(b-a\right)}^{2}}>0 \end{array}$$

so this function has two solutions given as follows:

(A10) $$r=\frac{-b\pm \sqrt{\Delta }}{2a}=\frac{-\frac{CDb+hQ}{QD\left(b-a\right)}\pm \frac{\sqrt{2ChDQ\left(b-a\right)+h^{2}Q^{2}}}{QD\left(b-a\right)}}{-\frac{C}{QD\left(b-a\right)}}=\frac{CDb+hQ\mp \sqrt{2ChDQ\left(b-a\right)+h^{2}Q^{2}}}{C}$$

First, we know $\sqrt{2ChDQ\left(b-a\right)+h^{2}Q^{2}}\leq CD\left(b-a\right)+hQ$. So $CDb+hQ-\sqrt{2ChDQ\left(b-a\right)+h^{2}Q^{2}}\geq CDb+hQ-CD\left(b-a\right)-hQ=CDa\geq 0$. So if $\;0<r<\frac{CDb+hQ-\sqrt{2ChDQ\left(b-a\right)+h^{2}Q^{2}}}{C}$, $\frac{\partial E\left[C\left(Q,r\right)\right]}{\partial r}<0$, E[C(Q,r)] is monotonically decreasing. If $\frac{CDb+hQ-\sqrt{2ChDQ\left(b-a\right)+h^{2}Q^{2}}}{C}<r<\frac{CDb+hQ+\sqrt{2ChDQ\left(b-a\right)+h^{2}Q^{2}}}{C}$, $\frac{\partial E\left[C\left(Q,r\right)\right]}{\partial r}>0$, *E*[*C*(*Q*,*r*)] is monotonically increasing. If $\frac{CDb+hQ+\sqrt{2ChDQ\left(b-a\right)+h^{2}Q^{2}}}{C}<r$, $\frac{\partial E\left[C\left(Q,r\right)\right]}{\partial r}<0$, E[C(Q,r)] is monotonically decreasing. So $\frac{CDb+hQ-\sqrt{2ChDQ\left(b-a\right)+h^{2}Q^{2}}}{C}$ is minimal value of E[C(Q,r)]. And $\frac{CDb+hQ+\sqrt{2ChDQ\left(b-a\right)+h^{2}Q^{2}}}{C}$ is maximal value of E[C(Q,r)]. With constraints on hand, we get the optimal ordering quantity and reordering point which can minimize the total costs. □

Proposition 2 can be proved as follows:
**Proof** **of Proposition 2.** In the following, we assume that the lead time follows Exponential distribution. That is:

(A11) $$g\left(t\right)=\{\begin{array}{c} \lambda e^{-\lambda t}\;t>0\\ 0\;t\leq 0 \end{array}\;(\lambda >0)$$

Then, we can transform Equation (15) into:

$$\begin{array}{l} Min\;Z=E\left[C\left(Q,r\right)\right]\\ \;=\frac{DA+C\int_{\frac{r}{D}}^{\frac{W}{fD}}\frac{{\left(r-Dt\right)}^{2}}{2\;}g\left(t\right)dt}{Q}+KD+\frac{hQ}{2}\int_{0}^{\frac{W}{fD}}g\left(t\right)dt-hD\int_{0}^{\frac{r}{D}}tg\left(t\right)dt\\ \;+h\int_{0}^{\frac{r}{D}}rg\left(t\right)dt\\ \;=\frac{DA+C\int_{\frac{r}{D}}^{\frac{W}{fD}}\frac{{\left(r-Dt\right)}^{2}}{2}\lambda e^{-\lambda t}dt}{Q}+KD+\frac{hQ}{2}\int_{0}^{\frac{W}{fD}}\lambda e^{-\lambda t}dt-hD\int_{a}^{\frac{r}{D}}t\lambda e^{-\lambda t}dt\\ \;+h\int_{a}^{\frac{r}{D}}r\lambda e^{-\lambda t}dt\\ \;=\frac{AD}{Q}+KD\\ \;+\left(\frac{DWC^{2}}{\lambda fQ}-\frac{2rDC^{2}}{\lambda Q}+\frac{C^{2}D^{2}}{Q\lambda^{2}}-\frac{rWC^{2}}{fQ}+\frac{C^{2}r^{2}}{Q}+\frac{C^{2}W^{2}}{2Qf^{2}}\right)\left(e^{-\frac{\lambda r}{D}}-e^{-\frac{\lambda W}{fD}}\right)\\ \;+\frac{hQ}{2}\left(1-e^{-\frac{\lambda W}{fD}}\right)-\frac{hD}{\lambda }\left(1-e^{-\frac{\lambda r}{D}}\right)+hr \end{array}$$

(A12) $$s.t.\{\begin{array}{c} \begin{array}{c} 98\%\leq \int_{0}^{\frac{r}{D}}\lambda e^{-\lambda t}\mathrm{dt}\leq 99\% \end{array}\\ \int_{0}^{S-\frac{Q}{D}}\lambda e^{-\lambda t}dt\geq 99\%\\ Q\geq r\\ Q,r,t\geq 0 \end{array}$$

(a) Solution This model can be expressed by:

$$\begin{array}{l} Min\;Z=E\left[C\left(Q,r\right)\right]\\ \;=\frac{AD}{Q}+KD+\frac{C^{2}}{Q}\left(\frac{DW}{\lambda f}-\frac{2rD}{\lambda }+\frac{D^{2}}{\lambda^{2}}-\frac{rW}{f}+r^{2}+\frac{W^{2}}{2f^{2}}\right)\left(e^{-\frac{\lambda r}{D}}-e^{-\frac{\lambda W}{fD}}\right)\\ \;+\frac{hQ}{2}\left(1-e^{-\frac{\lambda W}{fD}}\right)-\frac{hD}{\lambda }\left(1-e^{-\frac{\lambda r}{D}}\right)+hr \end{array}$$

(A13) $$s.t.\{\begin{array}{c} 98\%\leq 1-e^{-\frac{\lambda r}{D}}\leq 99\%\\ 1-e^{-\lambda \left(S-\frac{Q}{D}\right)}\geq 99\%\\ Q\geq r\\ Q,r,t\geq 0 \end{array}$$

The first derivative of total costs with respect to ordering quantity is:

(A14) $$\frac{\partial E\left[C\left(Q,r\right)\right]}{\partial Q}=-\frac{DA+C^{2}\left(\frac{DW}{\lambda f}-\frac{2rD}{\lambda }+\frac{D^{2}}{\lambda^{2}}-\frac{rW}{f}+r^{2}+\frac{W^{2}}{2f^{2}}\right)\left(e^{-\frac{\lambda r}{D}}-e^{-\frac{\lambda W}{fD}}\right)}{Q^{2}}+\frac{h}{2}\left(1-e^{-\frac{\lambda W}{fD}}\right)$$

With the first order condition, we obtain the optimal value of *Q* as follows:

(A15) $$-\frac{DA+C^{2}\left(\frac{DW}{\lambda f}-\frac{2rD}{\lambda }+\frac{D^{2}}{\lambda^{2}}-\frac{rW}{f}+r^{2}+\frac{W^{2}}{2f^{2}}\right)\left(e^{-\frac{\lambda r}{D}}-e^{-\frac{\lambda W}{fD}}\right)}{Q^{2}}+\frac{h}{2}\left(1-e^{-\frac{\lambda W}{fD}}\right)=0$$

(A16) $$Q^{*}=\sqrt{\frac{2DA+2C^{2}\left(\frac{DW}{\lambda f}-\frac{2rD}{\lambda }+\frac{D^{2}}{\lambda^{2}}-\frac{rW}{f}+r^{2}+\frac{W^{2}}{2f^{2}}\right)\left(e^{-\frac{\lambda r}{D}}-e^{-\frac{\lambda W}{fD}}\right)}{h\left(1-e^{-\frac{\lambda W}{fD}}\right)}}$$

If $0<Q<Q^{*}$, $\frac{\partial E\left[C\left(Q,r\right)\right]}{\partial Q}<0$, E[C(Q,r)] is monotonically decreasing. If $Q>Q^{*}$, $\frac{\partial E\left[C\left(Q,r\right)\right]}{\partial Q}>0$, E[C(Q,r)] is monotonically increasing, so $Q^{*}$ is minimal value of E[C(Q,r)]. □
